# Biochemical indexes and gut microbiota testing as diagnostic methods for *Penaeus monodon* health and physiological changes during AHPND infection with food safety concerns

**DOI:** 10.1002/fsn3.2873

**Published:** 2022-04-22

**Authors:** Tze Chiew Christie Soo, Subha Bhassu

**Affiliations:** ^1^ Animal Genetics and Genome Evolutionary Laboratory (AGAGEL) Department of Genetics and Microbiology Faculty of Science Institute of Biological Sciences University of Malaya Kuala Lumpur Malaysia; ^2^ Terra Aqua Laboratory Centre for Research in Biotechnology for Agriculture (CEBAR) Research Management and Innovation Complex University of Malaya Kuala Lumpur Malaysia

**Keywords:** 16S rRNA analysis, acute hepatopancreatic necrosis disease, biochemical tests, food safety, *Penaeus monodon*

## Abstract

Severe shrimp disease outbreaks have a destructive impact on shrimp aquaculture and its associated downstream food processing industries. Thus, it is essential to develop proper methods for shrimp disease control, which emphasizes the importance of food safety. In this study, we performed biochemical tests and gut microbiome analysis using uninfected control and *Vp*_AHPND_‐infected *Penaeus monodon* samples. Biochemical tests were performed to assess the phenoloxidase (PO) activity, respiratory Burst (RB) activity, nitrite concentration, superoxide dismutase (SOD) activity, total hemocyte count (THC), and total protein concentrations. Overall, upregulations were detected in these biochemical tests, which showed the activation of the immune response in *P. monodon* during acute hepatopancreatic necrosis disease (AHPND) infection, especially at 6 hpi and 12 hpi. Besides that, shrimp gut samples were collected and pooled (*n* = 3), followed by DNA extraction, PCR amplification targeting the V3/V4 16S ribosomal RNA (rRNA) region, next‐generation sequencing (NGS), and bioinformatics analysis. Proteobacteria was the most abundant phylum in both samples. The Rhodobacteraceae family and *Maritimibacter* genus were proposed to be vital forshrimp health maintenance. *Vp*_AHPND_ bacterial colonization and secondary *Vibrio* infections were postulated to have occurred based on the higher abundances of Vibrionaceae family and *Vibrio* genus in the *Vp*_AHPND_‐infected sample. Firmicutes phylum together with *Photobacterium* and *Aliiroseovarius* genera were inferred to be pathogenic or related factors of AHPND infections. In conclusion, physiology (immune response activation) and gut microbiome changes of disease tolerant *P. monodon* during AHPND infection were identified. Both biochemical tests and 16S rRNA analysis are proposed as a combined strategy for shrimp health diagnosis for ensuring shrimp health maintenance, disease control, and food safety.

## Graphical abstract

Severe shrimp disease outbreaks have a destructive impact on shrimp aquaculture and its associated downstream food processing industries. Thus, it is essential to develop proper methods for disease control, which emphasizes on the importance of food safety. In this study, we successfully performed biochemical tests and 16S gut microbiome analysis using uninfected control and V*p*_AHPND_‐infected *P. monodon* samples.

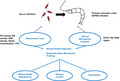

## 1 INTRODUCTION

The global shrimp aquaculture industry is a crucial source of aquatic food and protein, in which aquaculture shrimp production constituted more than half of the world's shrimp supplies since the year 2015 (Anderson et al., 2016). In Asia, shrimp production successfully achieved 3.75 million metric tons in the year 2018, with an estimated production of 4.00 million metric tons in the year 2021 (Anderson et al., 2019). However, shrimp diseases remain a major challenge for shrimp farmers (Anderson et al., 2019). Some recent important shrimp diseases are acute hepatopancreatic necrosis disease (AHPND), hepatopancreatic microsporidiosis, white spot disease, yellow head disease, hepatopancreatic haplosporidiosis, covert mortality disease, aggregated transformed microvilli, and infectious myonecrosis (Thitamadee et al., 2016).

Acute hepatopancreatic necrosis disease disease is a shrimp bacterial disease that can cause 40% to 100% mortality within the early 35 days of shrimp stocking (Hong et al., 2016). This disease is caused by a pathogenic strain of Gram‐negative *Vibrio parahaemolyticus* bacteria called *Vp*_AHPND_ (Lee et al., 2015; Tran et al., 2013). These *Vp*_AHPND_ bacteria are capable of producing *Photorhabdus* insect‐related (Pir) toxins, PirA and PirB, encoded by corresponding genes located within a 70‐kbp plasmid (pVA1) (Lee et al., 2015). Since its first emergence in China in the year 2009, AHPND had spread to Southeast Asia and Mexico regions, which caused significant reductions in shrimp aquaculture production (Fao, 2013; Nunan et al., 2014). The common gross clinical signs of AHPND infection are slow growth, empty gut, empty stomach, and pale or atrophied hepatopancreas (Hong et al., 2016). The shrimp species susceptible to AHPND infection include *Penaeus monodon*, *Litopenaeus vannamei*, and *Fenneropenaeus chinensis* (Zorriehzahra & Banaederakhshan, 2015).

Despite being one of the seafood that cause human illnesses globally (Bondad‐Reantaso et al., 2008), there had been insufficient studies on shrimp diseases from the perspective of food safety. The majority of shrimp‐related food safety studies were focused on dietary supplementation (Li et al., 2007) or the food processing (Kaur et al., 2013). Seafood‐borne illnesses are majorly caused by *Vibrio* species, especially *V. parahaemolyticus*, *V. cholerae*, and *V. vulnificus* (Bondad‐Reantaso et al., 2008; Gopal et al., 2005). The bacterial infections that are potentially pathogenic to humans may have occurred in diseased aquatic animals, particularly those caused by Vibrios (Austin, 2010). In a previous study, Gopal et al. (2005) showed that even though not all isolated *Vibrio* species possessed pathogenicity or toxicity traits, a significant percentage composition of these *Vibrio* species found and isolated from the farmed shrimp samples clearly exhibited hidden risks associated with food safety in shrimp consumption. Such hidden risks can be eliminated by ensuring proper shrimp health detection and shrimp gut microbiome monitoring.

The correct and accurate determination of shrimp physiological changes is vital for shrimp health detection and disease prevention. Physiological changes in shrimps in response to stress or diseases are usually detected through various biochemical tests. Common biochemical tests applied are for the estimation of phenoloxidase (PO) activity (Hsieh et al., 2008; Lin et al., 2011), respiratory burst (RB) activity (Lin et al., 2011), superoxide dismutase (SOD) activity (Hsieh et al., 2008; Lin et al., 2011), nitrite concentration (Zokaeifar et al., 2014), total protein (Santhoshkumar et al., 2017; Zokaeifar et al., 2014), and total hemocyte count (THC) (Hsieh et al., 2008; Lin et al., 2011).

Other than that, another essential strategy for shrimp health diagnosis and disease prevention would be shrimp gut microbiome analysis. This is because of the crucial roles played by gut microbiota in the various physiological processes, including metabolism (Tremaroli & Bäckhed, 2012), immune regulation (Maynard et al., 2012), endocrine function (Clarke et al., 2014), and pathogen elimination (Endt et al., 2010). Ever since the widescale application of high throughput next‐generation sequencing (NGS) technology, there had been increasing demands of NGS application in gut microbiome analysis especially involving 16S rRNA diversity due to cost and effectiveness concerns (Caporaso et al., 2011). 16S rRNA studies are usually conducted for the identification of microbial diversity related to host or environmental parameters (Marzinelli et al., 2015) and pattern of gene content (Konstantinidis & Tiedje, 2005). The V3/V4 hypervariable region of the 16S rRNA gene is commonly targeted for 16S rRNA sequencing analysis, as demonstrated in some previous works (Fan et al., 2019; Porchas‐Cornejo et al., 2017; Zoqratt et al., 2018). An advantage of 16S rRNA sequencing analysis would be its less reliance on the quality of extracted DNA samples (Rintala et al., 2017). Although in fewer frequencies, there were also some publications of 16S rRNA analysis involving healthy and diseased shrimps (Cornejo‐Granados et al., 2017; Liang et al., 2020; Zhou et al., 2019).

Therefore, this study aims to identify the physiology and gut microbiome changes of *P. monodon* during AHPND infection. The biochemical tests and 16S rRNA sequencing technique are also proposed as a combined enhanced strategy for the diagnosis of shrimp disease and the regulation of shrimp health, from the perspective of food safety and nutrition.

## 2 MATERIALS AND METHODS

### 2.1 Shrimp pathogenic challenge and sample collection

Juvenile disease tolerant crossbred *P. monodon* shrimps (5th generation Malaysian strain crossed with 13th generation Madagascar strain) with an average body length of 15–20 cm were collected from a local commercial farm. A modified immersion method (Tran et al., 2013) based AHPND bacterial challenge experiment was conducted as described previously (Devadas, 2019; Soo et al., 2019). Initial isolation, validation, incubation, and selective enrichment of the *Vp*_AHPND_ bacteria local strain, KS17.S5‐1 was done (Devadas et al., 2018). The incubation and selective enrichment of the *Vp*_AHPND_ bacteria were done using tryptic soy broth (TSB+) (2% sodium chloride supplemented) (Merck), thiosulfate citrate bile salt (TCBS) agar (Merck), and tryptic soy agar (2% sodium chloride supplemented) (Merck). The incubation condition utilized was 28°C for 18 h at 120 rpm. The obtained *Vp*_AHPND_ bacteria was verified using AP3 polymerase chain reaction (PCR) method (Sirikharin et al., 2015).

For the AHPND experimental challenge, *Vp*_AHPND_ bacteria (KS17.S5‐1 strain) at a concentration of 2 × 10^6^ cfu/ml was used for the *Vp*_AHPND_‐infected treatment group, whereas sterile TSB + broth was used for the uninfected control treatment group. 27 shrimps were placed in each tank filled with aerated artificial seawater (30 ppt) at 28 ± 1.0°C under an aseptic setup. Shrimp acclimatization was performed for 7 days before the challenge experiment. Three shrimp samples from each treatment group were collected at 0, 3, 6, 12, 24, 36, and 48 h post‐infection (hpi) and their vital organs (hepatopancreas, gut, muscle, and haemolymph) were stored at −80°C. The AHPND infection was validated by the observation of gross clinical symptoms (pale white hepatopancreas, empty stomach, and empty gut) and the AP3 PCR detection method (Sirikharin et al., 2015). The Ethical approval for this work was granted by the University of Malaya (Ethical Application Ref: S/31012019/26112018‐05/R).

### 2.2 Biochemical test validation

Hepatopancreas from the collected *P. monodon* samples were homogenized in 1X phosphate‐buffered saline (PBS) at a ratio of 1:9 and centrifuged at 13,300 g for 20 min at 4°C. The supernatant was collected from homogenized samples, diluted based on downstream applications, and stored at −20°C. The collected shrimp muscle and hemolymph samples were also utilized for biochemical analysis. The biochemical experiments were performed in a Greiner 96‐well U bottom microplate (Greiner Bio‐One).

All biochemical assay experiments were conducted with three biological replicates and three technical replicates for each. Statistical analysis involving one‐way analysis of variance (ANOVA) accompanied by Duncan's post hoc test was performed. The statistical significance value was set at *p* < .05. The raw data for the biochemical assay experiments was provided in Data S1.

#### 2.2.1 Bradford protein assay

Hepatopancreas and muscle samples of *P. monodon* were homogenized (mixed with 1X PBS at the ratio of 1:9 in ice water) and centrifuged (580 g for 10 min), and the supernatant was collection (diluted as and when needed) and utilized for the Bradford protein assay experiment. The total protein concentrations were identified by the Bradford Assay method (Bradford, 1976) with slight modifications. For the modified Bradford protein estimation assay, 5 µl of homogenized sample supernatant was mixed with 250 µl of 1X Bradford reagent (Coomassie brilliant blue G‐250 dissolved in methanol, phosphoric acid, and water) and was subsequently incubated for 50 min at room temperature. Then, the absorbance of the solution was measured at 595 nm, using a Tecan M200 Infinite Pro Microplate Reader (Tecan Group). The positive control used was bovine serum albumin (BSA) solution (for standard curve plotting), whereas the negative control used was 1X PBS.

#### 2.2.2 Phenoloxidase activity assay

Phenoloxidase activity assay was done by a previously described modified method (Hong et al., 2019; Park et al., 2019). The assay involved the transformation of L‐3, 4‐dihydroxyphenylalanine (L‐DOPA) to dopachrome. 50 µl of the homogenized hepatopancreas sample supernatant was mixed with 50 µl of 1X PBS and 50 µl of L‐DOPA (3 mg/ml) (dissolved in PBS). The dopachrome formation was then measured at 490 nm for every one min for a total time of 10 min using the Tecan M200 Infinite Pro Microplate Reader (Tecan Group). The PO activity was measured as the maximum change of A490 nm/min × 10^3^ per mg protein. One unit was defined as the increase of 0.001 A490 nm/min. The result normalization was done using the protein concentrations previously identified through the Bradford Assay method. 1X PBS solution was used instead of sample for the negative control.

#### 2.2.3 Respiratory burst activity assay

Respiratory burst activity assay was carried out based on a previously described method (Huynh et al., 2011) with slight modifications. The 96‐well microplate was coated with 100 µl of poly‐L‐lysine solution (0.2% v/v) (diluted with deionized water) 24 h before use for increased cell adhesion. 50 µl of diluted hemolymph samples were mixed with 100 µl of Zymosan A (1 mg/ml) in modified complete Hank's balanced salt solution (MCHBSS) and the mixture was left to react for 30 min at room temperature. 100 µl of nitro blue tetrazolium chloride (NBT) solution (0.3% w/v) was then added to the mixture and it was incubated for 30 min at room temperature. Then, 50 µl of 100% methanol was added to stop the reaction. The mixture was discarded, and the microplate was washed thrice with 100 µl of 70% methanol and air‐dried for 30 min. 120 µl of potassium hydroxide (KOH) (2 M) (dissolved in deionized water) and 140 µl of dimethyl sulfoxide (DMSO) were added for dissolving the insoluble formazan crystals formed from NBT reduction. The RB activity was measured at 630 nm using the Tecan M200 Infinite Pro Microplate Reader (Tecan Group) as the measurement of superoxide anion generation. Zymosan A solution in MCHBSS was used as the positive control, whereas 1X PBS was used as the negative control.

#### 2.2.4 Relative superoxide dismutase activity assay

The relative SOD activity was measured based on a method described by Perera et al. (2017) with slight modifications. Initially, the reaction mixture containing 20 µl of homogenized hepatopancreas sample supernatant or 1X PBS (negative control), 160 µl of glycine‐NaOH buffer (pH 9; 0.1 M) (dissolved in deionized water), and 6.75 µl of each: ethylenediaminetetraacetic acid (EDTA) (3 mM) (dissolved in deionized water), 0.15% BSA, xanthine (3 mM) (dissolved in deionized water), and NBT (0.75 mM) (dissolved in deionized water) was prepared. The reaction mixture was incubated for 10 min at room temperature. The reaction was then initiated by adding 6 mU of xanthine oxidase (dissolved in deionized water) and allowed to run for 20 min at room temperature. The SOD activity was measured at 560 nm every two mins for a total period of 20 min, using the Tecan M200 Infinite Pro Microplate Reader (Tecan Group). The relative SOD activity was calculated as the percentage of respective SOD enzyme activity from the highest SOD enzyme activity (100%).

#### 2.2.5 Nitrite concentration assay

The nitrite concentration measurement was estimated by a modified method based on previous publications (Al‐Amin et al., 2015, 2016; Tracey et al., 1995). 6 µl of homogenized hepatopancreas sample supernatant was mixed with 44 µl of deionized water and 20 µl of phosphate buffer (pH 7.5; 0.31 M) (dissolved in deionized water). The sample mixture was placed in the dark for one hour at room temperature. Subsequently, 200 µl of Griess reagent (1:1 mixture of sulfanilamide solution [1% sulfanilamide in 5% phosphoric acid] and NED solution [0.1% w/v N‐1‐naphthylethylenediamine dihydrochloride dissolved in deionized water]) was added. The assay was incubated in the dark at room temperature for another 10 min before being measured at 540 nm using the Tecan M200 Infinite Pro Microplate Reader (Tecan Group). A serially diluted 100 µM nitrite solution (sodium nitrite dissolved in deionized water) was used for the plotting of a nitrite standard reference curve. The A540 nm measurements of the samples were then converted to nitrite concentrations by matching the nitrite standard reference curve plotted. The negative control used was 1X PBS.

#### 2.2.6 Total hemocyte count

The THC was obtained using a modified standard method as described previously (Huynh et al., 2018). Hemolymph samples were mixed with 0.5% trypan blue solution (diluted with deionized water) at a dilution ratio of 1:1. The Neubauer improved hemocytometer (Marienfeld) and its coverslip were wiped with 70% ethanol before usage. The coverslip was set onto the hemocytometer, and 10 µl of diluted hemolymph mixture was filled into one of the chambers of the hemocytometer. The cells were counted under 40× magnification using Leica DM750 upright microscope (Leica Microsystems).

### 2.3 Gut microbiome analysis

#### 2.3.1 DNA extraction and 16S rRNA sequencing

DNA samples were extracted from the pooled *P. monodon* gut samples (*n* = 3 for each group) using the phenol‐chloroform DNA extraction method (Barker, 1998; PacBio, 2012) with modifications. The DNA extraction involved the initial cutting of 50–100 mg of sample into small pieces. The next step was the addition of 300 µl of lysis buffer (10 mM Tris‐HCl [pH 8], 25 mM EDTA [Merck], 100 mM NaCl, and 2% sodium dodecyl sulfate) (OIE, 2019), 18 µl of RNase A solution (10 mg/ml) (Bio Basic Inc.), and 18 µl of Proteinase K solution (20 mg/ml) (Bioteke Corporation), and then the solution was mixed by inversion and incubation in heat at 65°C for 2 h. 300 µl of phenol‐chloroform‐isoamyl alcohol (25:24:1) was then added, mixed, and centrifuged at 16,000 *g* for 5 min at 27°C to achieve 3 layers separation. 150 µl of the upper aqueous layer was removed and mixed with 300 µl of chloroform‐isoamyl alcohol (24:1), and then separated under the same previous centrifugation conditions. Cold precipitation was conducted at −80°C for 1 h using the newly separated 150 µl of the upper aqueous layer with the addition of 0.5 × volume of ammonium acetate and 2.5 × volume of 100% ethanol. This was followed by pellet formation (30 min centrifugation at 16,000 *g* and 4°C), and the pellet formed was washed twice with 150 µl of 70% ethanol (15 min centrifugation at 16,000 *g* and 4°C) and air dried (5–10 min), and the final DNA elution done using 50 µl of TE buffer. The supernatant was discarded for the steps before the final elution. The concentrations and qualities of the extracted DNA samples were checked using a NanoDrop™ 2000/2000c Spectrophotometer (Thermo Fisher Scientific).

The targeted V3/V4 region of the 16S rRNA gene was amplified through PCR using the condition of initial denaturation of 95°C for 2 min, followed by 25 cycles of 95°C for 30 s, 55°C for 30 s, 72°C for 30 s, and final extension of 72°C for 5 min. The PCR mixtures used involved 4 μl of 5 × FastPfu Buffer, 2 μl of 2.5 mM dNTPs, 0.8 μl of each primer (5 μM), 0.4 μl of FastPfu polymerase, and template DNA. The PCR products were then verified with 2% agarose gel electrophoresis, purified using AxyPrep DNA Gel Extraction Kit (Axygen Biosciences) according to the manufacturer's instructions, and quantified using QuantiFluor™ ‐ST (Promega). The library was then constructed that involved “Y” adapter linkage, bead‐based adapter dimer removal, PCR amplification for library concentration, and generation of single‐stranded DNA fragments using sodium hydroxide. Finally, the sample libraries were pooled in equimolar concentration and were paired‐end sequenced (2 × 250/300 bp) with the help of the Illumina MiSeq platform based on the standard protocol.

#### 2.3.2 Bioinformatics analysis and statistical validation

The quality of the raw data sequences was checked using FastQC software (Andrews, 2010) and Usearch (Edgar, 2010) software. The sequences were then merged and trimmed using Vsearch software (Rognes et al., 2016) and Trimmomatic software (Bolger et al., 2014) (Average quality score of 20, 50 bp minimum length, 50 bp sliding window). The trimmed sequences obtained were clustered into operational taxonomic units (OTUs) using UPARSE software (Edgar, 2013) with a 97% similarity cut‐off. The chimera sequences were removed using Usearch software (Edgar, 2010). The 16S rRNA OTUs obtained were taxonomically classified using RDP Classifier (Wang et al., 2007) by matching against Silva (SSU123) 16S rRNA database (confidence threshold of 0.7). The microbial communities of the sequenced samples were plotted in bar charts at phylum, family, and genus levels. Analysis with the Linear discriminant analysis effect size (LEfSe) tool was carried out using OTUs obtained to identify the differentially abundant bacterial taxa up to the genus level (LDA cutoff value: 5.0 or higher) (Segata et al., 2011). The alpha diversity parameters, including Good's coverage, Chao 1 estimator, Shannon index, and Simpson index, were calculated using Mothur software (Schloss et al., 2009). The alpha diversity parameters (Chao 1 estimator, Shannon index, and Simpson index) were further plotted as box plot diagrams using R software (Team RC, 2013). Rarefaction and Shannon rarefaction curves were determined and plotted using Mothur software (Schloss et al., 2009) and R software (Team RC, 2013). The beta diversity parameters, including Bray‐Curtis dissimilarity, Euclidean distance, Jaccard coefficient, and Manhattan distance were also calculated and plotted as PCOA diagrams using Usearch software (Edgar, 2010) and R software (Team RC, 2013). The raw data sequences (healthy and infected samples) of previous studies (Foysal et al., 2021; Hossain et al., 2021) were retrieved from NCBI database (BioProject ID: PRJNA662111; PRJNA662500) and used in the beta diversity analysis for cross comparison purpose. A Venn diagram was plotted using Mothur software (Schloss et al., 2009) and R software (Team RC, 2013).

#### 2.3.3 Data availability

The 16S rRNA analysis raw data is available on the NCBI Sequence Read Archive (SRA) database (SRA Accession Number: SRR12199297; SRR12199298) (BioProject ID: PRJNA645513) (BioSample ID: SAMN15507524; SAMN15507525).

## 3 RESULTS

### 3.1 Detection of activated immune response through biochemical tests

**FIGURE 1:**
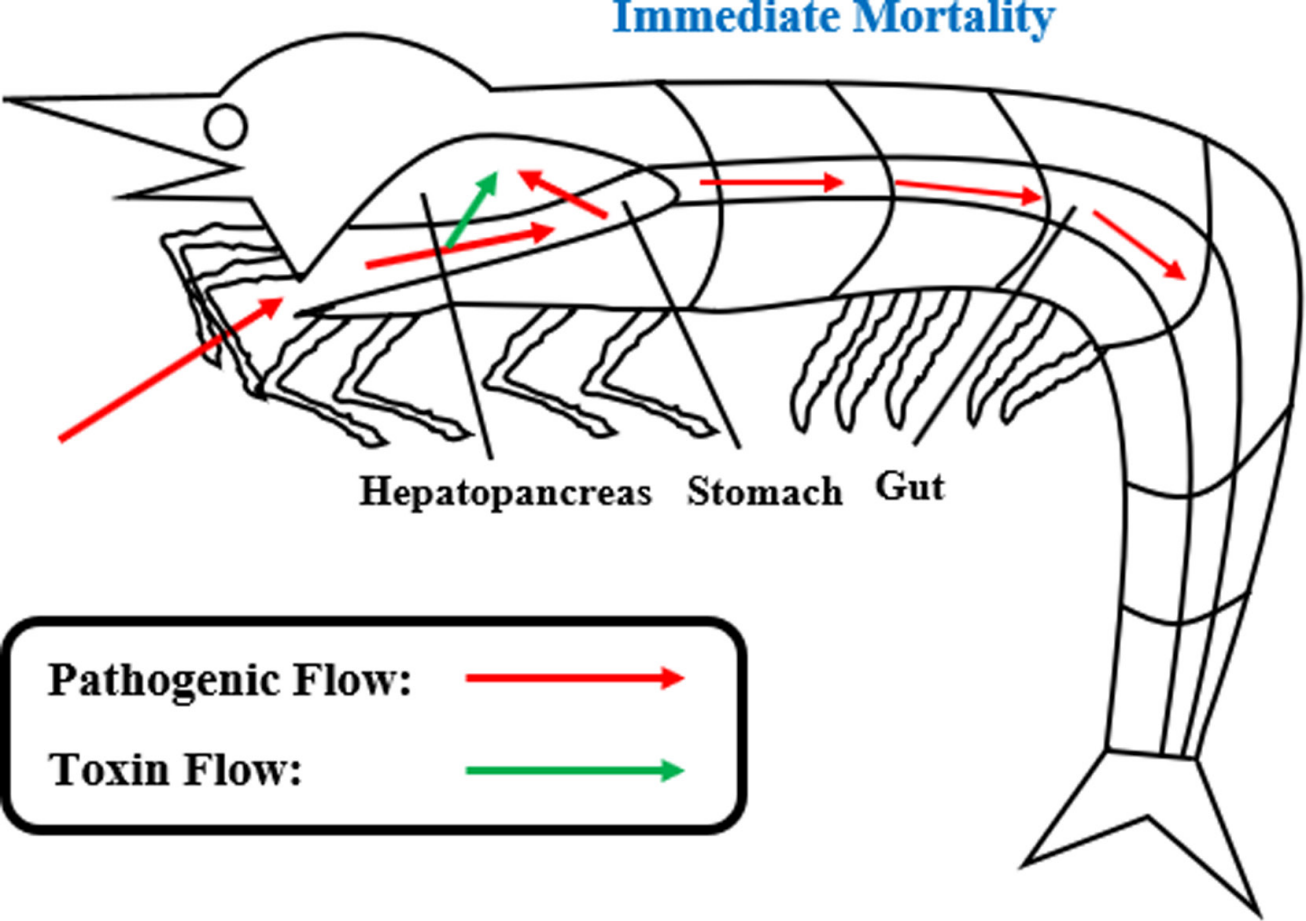
Overall pathogenic and toxin flow of AHPND infection in *Penaeus monodon*

Similar to previous work (Pan et al., 2008), the activated immune response of disease tolerant *P. monodon* during AHPND infection can be detected through suitable key immune parameters such as biochemical‐related ones. In this study, several biochemical tests including, PO activity assay, RB activity assay, nitrite concentration assay, relative SOD activity assay, and THC, were carried out and they successfully showed the differential biochemical activities associated with immune response activation of *P. monodon* during AHPND infection. The biochemical test results are presented in Table 1 and Figures [Link], [Link], [Link], [Link], [Link], [Link]. Statistical validations were also conducted through one‐way ANOVA (*p* < .05) and post hoc Duncan's test as shown in Tables [Link], [Link], [Link], [Link], [Link]. The Bradford protein assay was also performed for validation or normalization purposes. The Bradford protein assay results and subsequent statistical validation using one‐way ANOVA (*p* < .05) and post hoc Duncan's test are provided in Figures [Link], [Link], [Link] and Tables S6 and S7. The overall pathogenic and toxin flow of AHPND infection was also shown in Figure 1.

**TABLE 1:** Differential biochemical activities detected at different post‐infection time points of *Vp*_AHPND_‐infected *Penaeus monodon* shrimps

Hours post‐infection (hpi)	PO activity (U/total mg protein)	RB activity (OD 630 nm)	Average relative SOD activity (%)	Nitrite concentration (nmol/ml)	THC (×10^5^ ml^−1^)
Control	2.069 ± 0.0963	0.0483 ± 0.0144	68.83 ± 8.04	42.944 ± 9.316	4.778 ± 0.755
0	4.375 ± 1.142	0.0585 ± 0.0148	72.98 ± 25.47	33.278 ± 11.505	5.250 ± 1.710
3	3.483 ± 0.806	0.0498 ± 0.0106	81.85 ± 16.80	34.148 ± 14.492	3.878 ± 1.922
6	4.004 ± 0.869	0.0449 ± 0.0101	100.00 ± 27.26	55.185 ± 12.086	3.867 ± 0.404
12	14.664 ± 3.177	0.0781 ± 0.00535	38.50 ± 13.58	88.389 ± 13.455	4.161 ± 0.857
24	11.807 ± 3.016	0.0763 ± 0.00990	26.26 ± 16.21	54.796 ± 10.278	1.961 ± 0.351
36	6.237 ± 2.063	0.0397 ± 0.0188	47.00 ± 11.46	43.296 ± 14.500	1.300 ± 0.304
48	6.448 ± 3.886	0.0288 ± 0.00496	35.03 ± 18.87	48.000 ± 7.581	2.272 ± 0.300
PTC	—	0.0661 ± 0.00552	—	—	—

Note

The standard deviations (SD) were presented as ±SD values. All measured biochemical test results were statistically significant (*p* < .05).

Abbreviations: Control, uninfected control; PO, phenoloxidase; PTC, positive control; RB, respiratory burst; SOD, superoxide dismutase; THC, total hemocyte count.

Phenoloxidase activity was generally upregulated during AHPND infection with the highest peak identified at 12 hpi. The sharp upregulation of RB activity was detected at 12 hpi and 24 hpi by which the highest point was found at 12 hpi. The RB activity was then downregulated at 36 hpi and 48 hpi. In addition, SOD activity was stably upregulated until its highest peak at 6 hpi. However, it was then drastically downregulated at 12 hpi. Nitrite concentrations showed initial increment until the highest point at 12 hpi and were downregulated from 24 hpi to 48 hpi. The THC results were generally reduced by which the lowest point was detected at 36 hpi. Compared to the stable total protein concentration of *Vp*_AHPND_‐infected *P. monodon* hepatopancreas samples with no significant changes, the total protein concentration of *Vp*_AHPND_‐infected *P. monodon* muscle samples showed a significant decrease from 0 hpi to 48 hpi with the lowest point identified at 36 hpi. Overall, based on the biochemical tests results, the immune response activation of disease tolerant *P. monodon* was mainly detected at early AHPND infection period, particularly at 6 hpi and 12 hpi.

### 3.2 General analysis and quality assessment of 16S rRNA sequencing

**FIGURE 2:**
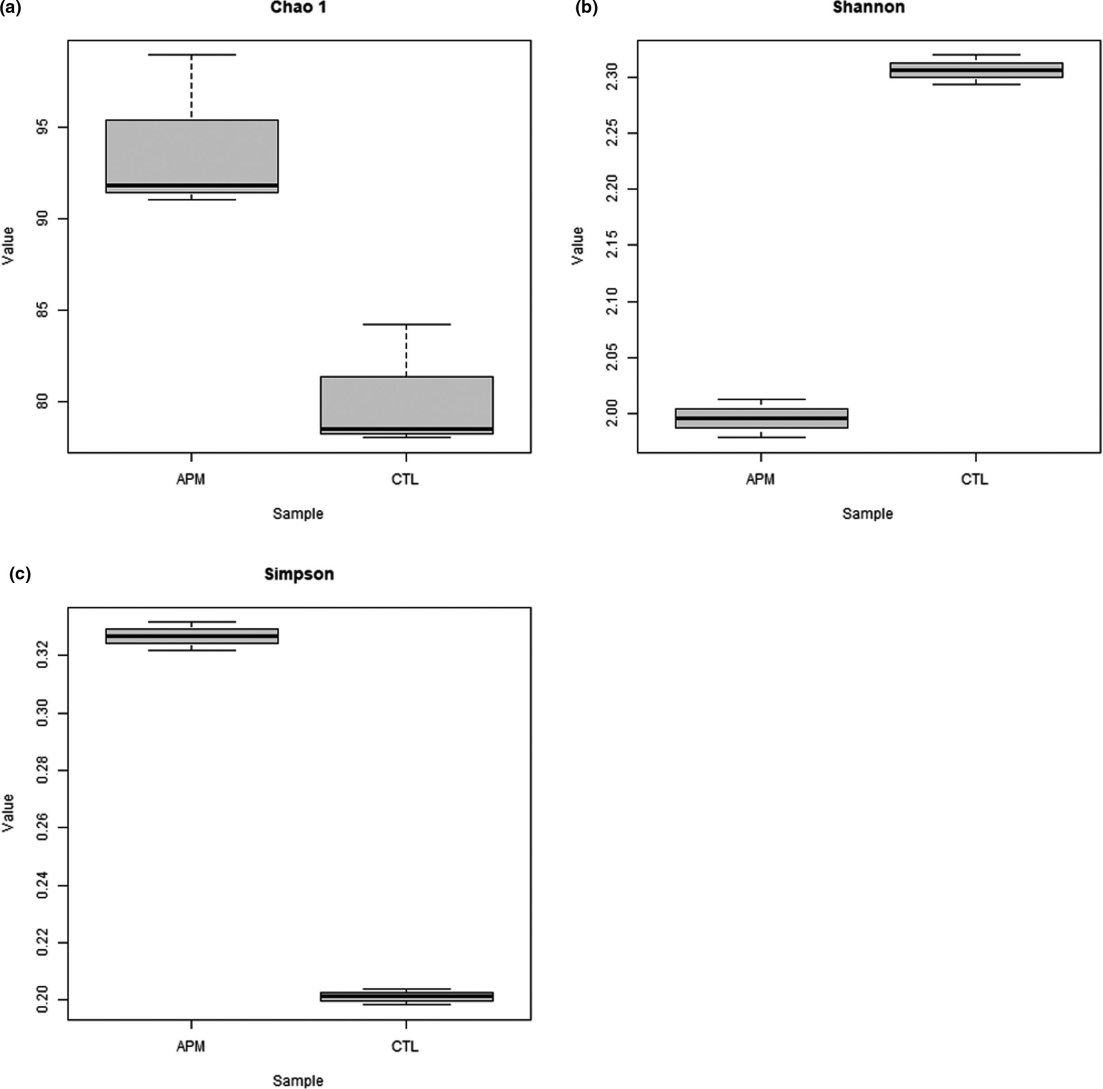
The alpha diversity parameters of 16S rRNA sequencing data: (a) Chao 1, (b) Shannon, (c) Simpson. APM: *Vp*_AHPND_‐infected; CTL: uninfected control

**FIGURE 3:**
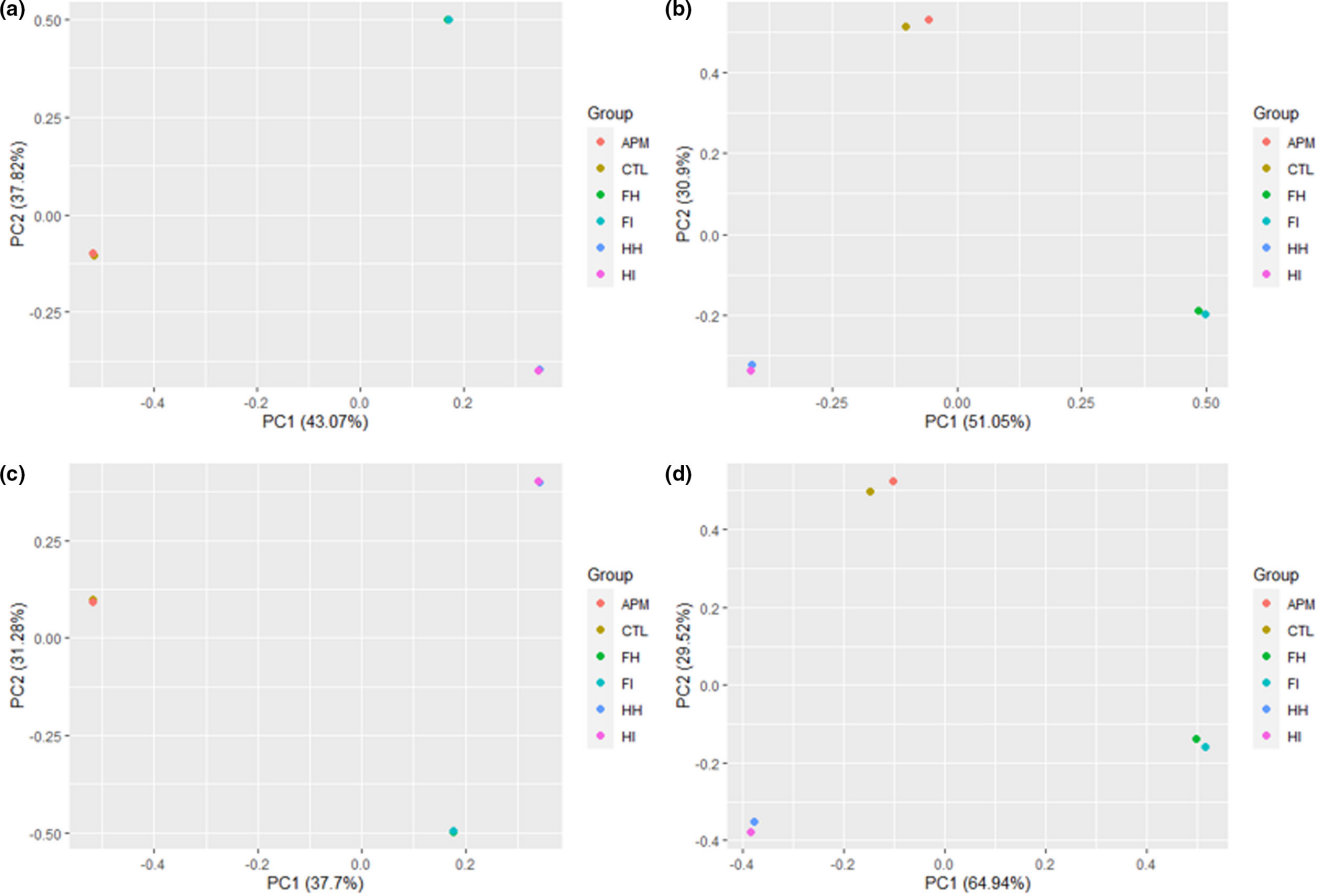
The beta diversity parameters of 16S rRNA sequencing data: (a) Bray–Curtis Dissimilarity, (b) Euclidean distance, (c) Jaccard coefficient, (d) Manhattan distance. APM: *Vp*_AHPND_‐infected; CTL: uninfected control; FH: Foysal et al. (2021) healthy; FI: Foysal et al. (2021) infected; HH: Hossain et al. (2021) healthy; HI: Hossain et al. (2021) infected

From the 16S rRNA sequencing analysis conducted, a total of 102,455 raw reads and 95,556 clean reads were obtained followed by 99.95% of operational taxonomic units (OTUs) determined to possess sequence lengths of 401–450 bp. The 16S rRNA sequencing data quality was further validated through several alpha diversity parameters as demonstrated in Figure 2, supported by Figures S10 and S11 together with Table S8. The Good's coverage values were 99.99% (0.9999) for both samples, which signified sufficient sequencing depth. In addition, the *Vp*_AHPND_‐infected sample demonstrated higher values in Chao 1, Shannon, and Simpson indexes compared to the uninfected control sample, which suggested greater levels of bacterial richness and diversity. Furthermore, beta diversity analysis was conducted as shown in Figure 3. The healthy and infected sample groups were clustered accordingly with greater distance observed between uninfected control and *Vp*_AHPND_‐infected sample groups compared to other sample groups in Euclidean distance and Manhattan distance of the beta diversity analysis (Figure 3). The sample groups of the current study were clustered apart from those of previous studies (Figure 3). Besides that, a Venn diagram showing the OTU distribution between the uninfected control and *Vp*_AHPND_‐infected samples was plotted, as shown in Figure S12. From the total OTUs (60), the number of unique OTUs was higher in the *Vp*_AHPND_‐infected sample (26) compared to the uninfected control sample, (16) whereas the number of overlapping OTUs was 18.

### 3.3 Overall comparison of gut microbiota diversity (relative abundance) of uninfected control and *Vp*_AHPND_‐infected shrimps

**FIGURE 4:**
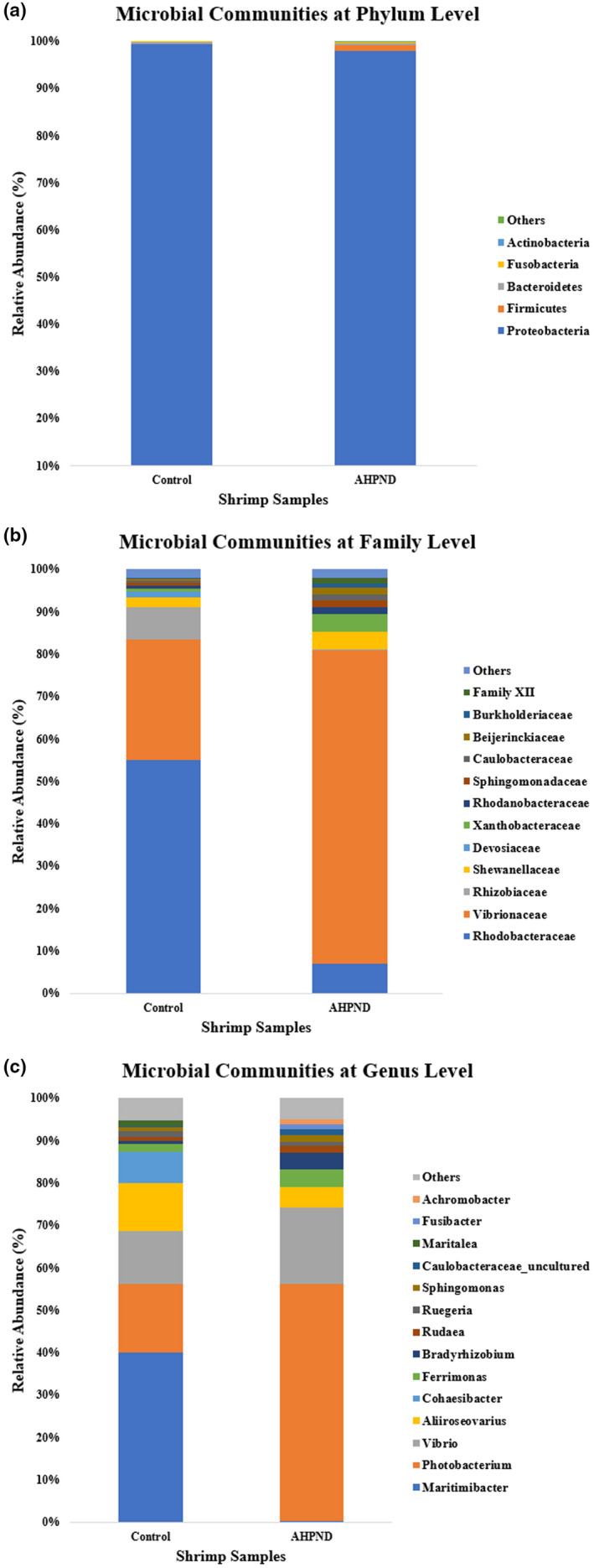
Microbial communities of uninfected control (Control) and *Vp*_AHPND_‐infected (AHPND) *Penaeus monodon* gut samples at (a) Phylum, (b) Family, and (c) Genus levels

The microbiota diversity or relative abundance of OTUs (phylum, family, and genus levels) between uninfected control and *Vp*_AHPND_‐infected *P. monodon* gut samples were determined by 16S rRNA sequencing analysis as shown in Figure 4a‐c. There were 8 phyla in the uninfected control sample and 9 phyla in the *Vp*_AHPND_‐infected sample. The most abundant phylum for both groups in this study was Proteobacteria by which the percentages were 99.37% and 97.87% for uninfected control and *Vp*_AHPND_‐infected samples respectively. Intriguingly, Firmicutes phylum showed a near 28‐fold relative abundance increment from the uninfected control sample (0.046%) to the *Vp*_AHPND_‐infected sample.

**FIGURE 5:**
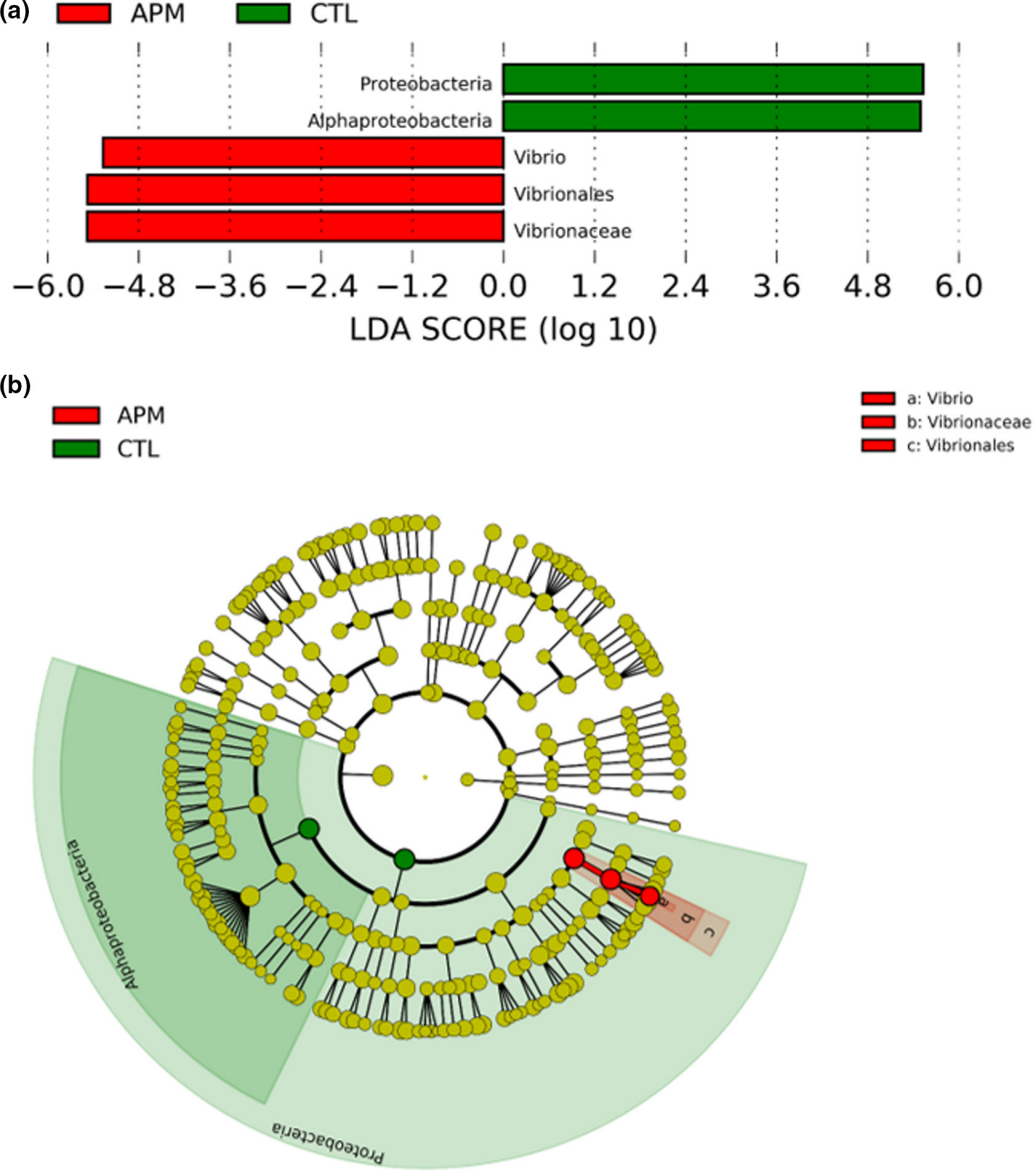
Linear discriminant analysis effect size (LEfSe) analysis conducted using OTUs obtained: (a) Differentially abundant taxa among compared sample groups based on computed Linear discriminant analysis (LDA) scores (LDA cutoff value: 5.0 or higher), (B) Circular cladogram plotted using LEfSe analysis (the dots represent operational taxonomic units [OTUs] at different taxonomic levels from center phylum level to outer circle genus level). APM: *Vp*_AHPND_‐infected; CTL: uninfected control

At family and genus levels, Rhodobacteraceae (55.0%), Vibrionaceae (28.6%), and Rhizobiaceae (7.5%) families together with *Maritimibacter* (40.1%), *Photobacterium* (16.1%), and *Vibrio* (12.4%) genera had the highest abundances in the uninfected control sample. On the other hand, Vibrionaceae (74.0%), Rhodobacteraceae (7.0%), and Shewanellaceae (4.1%) families together with *Photobacterium* (56.0%), *Vibrio* (17.9%), and *Aliiroseovarius* (4.8%) genera had the highest abundances in *Vp*_AHPND_‐infected sample. Vibrionaceae demonstrated a significant increase of relative abundance from the uninfected control sample (28.6%) to the *Vp*_AHPND_‐infected sample (74.0%), whereas Rhodobacteraceae showed a significant decrease of relative abundance from the uninfected control sample (55.0%) to the *Vp*_AHPND_‐infected sample (7.0%). At the genus level, the relative abundances of *Maritimibacter*, *Aliiroseovarius*, and *Cohaesibacter* in the *Vp*_AHPND_‐infected sample (0.21%; 4.8%; 0.10%) were significantly reduced compared to the uninfected control sample (40.1%; 11.3%; 7.4%). There was also a significant increment of the relative abundances of *Photobacterium*, *Vibrio*, and *Bradyrhizobium* from the uninfected control sample (16.1%; 12.4%; 0.69%) to the *Vp*_AHPND_‐infected sample (56.0%; 17.9%; 3.9%). Besides that, based on the linear discriminant analysis Effect Size (LEfSe) analysis results (Figure 5), Proteobacteria and Alphaproteobacteria were differentially abundant in uninfected control group whereas Vibrionales, Vibrionaceae, and *Vibrio* were differentially abundant in *Vp*_AHPND_‐infected group.

## 4 DISCUSSION

### 4.1 Immune response activation during AHPND infection

In this study, a series of biochemical tests (Table 1) using hepatopancreas, muscle, or haemolymph samples of uninfected control and *Vp*_AHPND_‐infected *P. monodon* shrimps was conducted. These biochemical tests successfully validated the immune response activation during AHPND infection. The immune response activation was shown by the upregulated PO activity, relative SOD activity, nitrite concentration, and RB activity during early post‐AHPND infection time points.

The upregulation of PO activity led to a stronger melanization response as melanization is controlled by the phenoloxidase enzyme activity in the prophenoloxidase (proPO) activation system (Alvarez & Chung, 2013; Amparyup et al., 2013; Hong et al., 2019; Park et al., 2019). There was a similar case of PO activity increment in *M. rosenbergii* after bacterial infection (Sung et al., 2004). The upregulation of RB and SOD activities inferred an elevated level of superoxide anion that caused increased antioxidation activity during AHPND infection. The elevation of nitrite concentration can trigger the activation of metabolic and immune reactions. In addition, nitrite formation in the redox reaction between nitric oxide and reactive oxygen species is vital for the elimination of pathogen through phagocytosis (Wink et al., 2011). However, excessively high nitrite concentration could cause adverse effects, including suppressed immune response, increased cytotoxic level, elevated susceptibility to bacterial infection, and increased superoxide anion level (Tseng & Chen, 2004).

The occurrence of hemocyte depletion due to apoptotic activity was also inferred through the decrease of THC levels. A similar scenario would be the occurrence of hemocyte depletion in *Drosophila* due to apoptotic activity that caused pro‐inflammatory state formation and alteration of the immune effector pathway (Arefin et al., 2015). Besides that, the general reduction of total protein concentrations in *Vp*_AHPND_‐infected *P. monodon* muscle samples, especially at 36 hpi, inferred protein degradation in shrimp muscle and probable muscle disturbance or degradation during AHPND infection. The inference was done based on a similar previous study that showed reduced total protein concentrations in both muscle and hepatopancreas of WSSV‐infected *Penaeus indicus* (Yoganandhan et al., 2003).

Furthermore, the importance of PO, SOD, and RB activities together with THC in the detection of shrimp immune response activation was highlighted in previous studies for either activated (Tayag et al., 2010) or suppressed (Li et al., 2008) immunity. As shown in a previous *Litopenaeus vannamei* challenge using *Vibrio harveyi* (Huang et al., 2013), despite the common reduction of SOD activity, THC, and PO activity during early infection time points, increased levels of SOD activity, THC, and PO activity were detected in disease‐resistant shrimps compared to non‐resistant shrimps. The elevated biochemical measurements were also accompanied by a faster recovery rate (Huang et al., 2013). Other than that, the immune humoral parameters involved, such as THC, PO, and NBT reduction, had been previously proposed as good potential stress indicators for aquatic animal health status (Verghese et al., 2007). Hence, this suggests the suitability of the biochemical parameters tested in this study as indicators of shrimp health status and immune response activation during pathogenic infection.

### 4.2 Gut microbiome changes during AHPND infection

Based on the alpha diversity parameters obtained (Figure 2 and Table S8), Good's coverage values of more than 0.99 (99%) identified for both uninfected control and *Vp*_AHPND_‐infected samples suggested satisfactory sequencing depth (Shin et al., 2019) and thus able to represent all bacterial communities. The higher alpha diversity parameter values determined in the *Vp*_AHPND_‐infected sample compared to the uninfected control sample showed stronger bacterial richness and diversity caused by AHPND infection. These alpha diversity parameters included Chao 1 richness estimation (Chao, 1984; Colwell & Coddington, 1994; Gotelli & Colwell, 2011) together with Shannon (Shannon, 1948) and Simpson (Simpson, 1949) diversity indices. The plateau stage was reached as the number of reads increased in the rarefaction (Figure S10) and Shannon rarefaction (Figure S11) curves plotted, which showed good sequencing depth and coverage (Liu, Yang, et al., 2020; Zhang et al., 2020). In general, the sequencing depth, coverage, richness, and diversity of the 16S data were successfully validated through all the alpha diversity parameters calculated.

From the microbiome relative abundances compared between uninfected control and *Vp*_AHPND_‐infected gut samples (Figure 4), the Proteobacteria phylum was most abundantly found in both samples. Similar relative abundances of Proteobacteria phylum were previously reported that involved cultured healthy (69.3%) and cultured *Vp*_AHPND_‐infected (70.2%) *L. vannamei* shrimps (Cornejo‐Granados et al., 2017). Notably, the Firmicutes phylum, which had a near 28‐fold relative abundance increment from the uninfected control sample to *Vp*_AHPND_‐infected sample, can be suggested as a potential determinant factor in the colonization or infection process of *Vp*_AHPND_ bacteria. Firmicutes bacteria can survive in different environments, including extreme conditions, and produce endospores (Galperin, 2016). Firmicutes bacteria was previously reported to have elevated gut relative abundance in *V. alginolyticus*‐infected crab (Shi et al., 2019) and a positive correlation of its gut relative abundance with immune gene expressions in dietary supplemented *Fenneropenaeus merguiensis* (Liu, Zhou, et al., 2020).

The Rhodobacteraceae family possessed the highest relative abundance in the uninfected control sample. Rhodobacteraceae family are marine bacteria that possess functional importance in sulfur and carbon biogeochemical cycling together with microorganism or macroorganism symbiosis (Pujalte et al., 2014). The significantly decreased Rhodobacteraceae family relative abundance in the *Vp*_AHPND_‐infected sample was inferred to be the effect of *Vp*_AHPND_ bacterial colonization and subsequently disrupted microbiome symbiosis during AHPND infection. At the genus level, for the uninfected control sample, *Maritimibacter* had the highest relative abundance and thus may play an essential role in the upkeep of normal healthy *P. monodon* gut microbiome balance. However, there are insufficient studies on this genus by which only two species were found at the current moment (Lee et al., 2007; Zhong et al., 2015).

In addition, the Vibrionaceae family and *Vibrio* genus showed higher abundances in the *Vp*_AHPND_‐infected sample compared to the uninfected control sample. These higher abundances were postulated to be caused by *Vp*_AHPND_ bacterial colonization and related to secondary *Vibrio* infections. The inhabitation of *Vibrio* spp. bacteria in the shrimp intestine was due to its chitin‐rich environment identical to other crustaceans (Sugita & Ito, 2006). The secondary bacterial infections that occurred were mentioned previously (Santos et al., 2020). The *Photobacterium* genus bacteria had the highest relative abundance in the *Vp*_AHPND_‐infected sample that may be correlated to secondary luminous bacterial infections (Prayitno & Latchford, 1995). *Aliiroseovarius* genus can be proposed as pathogenic bacteria related to *Vibrio* bacteria based on a previous decrement of relative abundances for *Vibrio* and *Aliiroseovarius* genera bacteria in *L. vannamei* treated with beneficial seaweed feeding (Elizondo‐González et al., 2020).

In the process of AHPND infection, *Vp*_AHPND_ bacteria started to colonize the shrimp's stomach after entry through the oral route. The bacteria then released PirA and PirB toxins, which led to damaging of the shrimp hepatopancreas. There was also the identification of both *Vp*_AHPND_ bacteria and its toxins in the shrimp hepatopancreas during later post‐infection time points (Lai et al., 2015). Additionally, the occurrence of *Vp*_AHPND_ bacterial colonization and hemocytic infiltration at the shrimp anterior midgut were detected during post‐AHPND infection time points (Soonthornchai et al., 2016). The *Vp*_AHPND_ bacterial colonization locations were also shown by the common use of shrimp digestive organs such as stomach, hepatopancreas, midgut, and hindgut in AHPND diagnosis (Zorriehzahra & Banaederakhshan, 2015).

Gut microbiota is vital in the gut immune response such that disrupted microbiota will lead to immune dysregulation (Round & Mazmanian, 2009). A balanced gut microbiome composition is crucial in disease control (Biesebeke, 2018; Buttó & Haller, 2016). The shrimp gut microbiota dysbiosis is correlated to disease severity, which also involves environmental stress factors (Xiong et al., 2015). A balanced composition of shrimp gut microbial communities can be maintained or enhanced using different methods, such as shrimp feed composition (Huang et al., 2016; Jescovitch et al., 2018; Landsman et al., 2019; Li et al., 2018; Ringø et al., 2016), microbiota supplementation (prebiotics and probiotics) (Butt et al., 2021; Holt et al., 2020; Vargas‐Albores et al., 2017), and water quality assessment (Bentzon‐Tilia et al., 2016). 16S rRNA sequencing technique had been previously applied in the selection of beneficial indigenous (natural marine environment) microbial communities (Vargas‐Albores et al., 2017) and microbes with disease inhibitory potential (Wanka et al., 2018) for probiotic supplementation. In general, the important *P. monodon* gut microbiome changes due to AHPND infection were identified through the 16S rRNA sequencing technique in this study.

### 4.3 Diagnostic and food safety applications

In this study, biochemical tests and 16S rRNA analysis are suggested as suitable diagnostic tools for the determination of shrimp health status and gut microbiome changes, especially between healthy and diseased shrimps. Such applications are vital for ensuring food safety in downstream consumption. Some of the commonly investigated biochemical aspects of food safety include biochemical lesions, enzyme inhibition, and congenital metabolic disorders (Walker, 1980). Biochemical tests were utilized in food safety studies, such as bacteria biochemical tests (ALatawi et al., 2015) and immunological biochemical tests (Sun et al., 2011). The common applications of immunological biochemical tests in food safety‐related studies would be probiotics or dietary supplementation works (Gupta et al., 2014; Kumar et al., 2013; Sun et al., 2011), challenged shrimp works (Vaseeharan et al., 2013), and combined works (Citarasu et al., 2006; Gholamhosseini et al., 2020). However, there had been a lack of attention and effort in connecting immunological biochemical tests of shrimp challenge works to food safety‐oriented applications.

On the other hand, due to the time and labor constraints of traditional food microbiology detection methods (Rodríguez‐Lázaro et al., 2007), PCR had risen to become the standard rapid detection method for food microbes (Hameed et al., 2018). 16S rRNA analysis either through conventional PCR (ALatawi et al., 2015) or real‐time qPCR (Wolffs et al., 2004) had been previously utilized in food safety studies. Furthermore, the importance of cultured environment bacterial composition and associated shrimp gut microbiota changes was successfully highlighted from the beneficial changes in growth, immune response, survival, and gut microbiome of probiotics‐supplemented *P. indicus* cultured under biofloc system (Panigrahi et al., 2020). Such importance suggests the necessity of 16S rRNA analysis to be applied in the diagnosis of shrimp health and detection of shrimp disease outbreaks. The shrimp health diagnosis can be achieved through the close comparison of shrimp gut relative abundances, particularly focusing on potentially pathogenic or disease‐related microbes such as *Vibrio* genera.

## 5 CONCLUSION

In conclusion, the biochemical tests performed in this study, including the estimation of PO activity, RB activity, SOD activity, nitrite concentration, and THC, successfully demonstrated *P*. *monodon's* immune response activation at 6 hpi and 12 hpi of the post‐AHPND infection time points. In addition, from the 16S rRNA analysis, microbial communities of the Rhodobacteraceae family and *Maritimibacter* genus were postulated to be important for shrimp health maintenance. On the other hand, potential AHPND‐related pathogenic factors determined would be the Firmicutes phylum, Vibrionaceae family, and *Photobacterium*, *Vibrio*, and *Aliiroseovarius* genera. The occurrence of secondary *Vibrio* infections associated with *Vp*_AHPND_ bacterial colonization was also suggested. Overall, the physiology and gut microbiota changes of *P. monodon* in response to AHPND infection were successfully determined using biochemical tests and the 16S rRNA sequencing technique. Hence, both biochemical tests and the 16S rRNA sequencing technique involved in this study are proposed as a combined strategy to be applied in ensuring shrimp health status diagnosis and disease control. The successful application of such strategies can then lead to stronger food safety and nutrition starting from the beginning of the food processing chain. Shrimp diseases are potentially accompanied by pathogens that are harmful to humans. Healthy shrimps not only are safer for consumption but also possess higher nutrition values compared to diseased shrimps.

Based on the results obtained, enhanced biochemical tests can be developed to achieve cost‐effective and efficient detection of shrimp health status changes, followed by the establishment of a biochemical‐based profiling system. The important beneficiary shrimp gut microbial communities identified in this study can assist in the development of enhanced shrimp supplements to achieve better shrimp health conditions and stronger disease resistance or tolerance. Other than that, an associated shrimp gut microbial profiling system can also be established for shrimp health diagnosis, disease detection, disease severity estimation (Dai et al., 2020; Xiong et al., 2015), and identification of potential polymicrobial infections (Dai et al., 2018).

## CONFLICT OF INTEREST

No conflict of interest is declared.

## Supporting information

Data S1

Figure S1

Figure S2

Figure S3

Figure S4

Figure S5

Figure S6

Figure S7

Figure S8

Figure S9

Figure S10

Figure S11

Figure S12

Table S1

Table S2

Table S3

Table S4

Table S5

Table S6

Table S7

Table S8
