# Supplementary material for: Biochemical indexes and gut microbiota testing as diagnostic methods for Penaeus monodon health and physiological changes during AHPND infection with food safety concerns
Source: Food Sci Nutr. 2022 Apr 22;10(8):2694–709. doi: 10.1002/fsn3.2873 (PMC9361443; doi:10.1002/fsn3.2873)
Supplement: Supplementary file 1 — Data S1 [file FSN3-10-2694-s008.docx]

**Data 1 Supp: Raw data of biochemical tests conducted.**

| **Groups** | **PO** | **RB** | **SOD** | **Nitrite** | **THC** | **Bradford** | |
| --- | --- | --- | --- | --- | --- | --- | --- |
|  |  |  |  |  |  | **HP** | **Muscle** |
| **C-1** | 0.00270 | 0.0362 | 0.00138 | 0.0275 | 20.583 | 0.492 | 1.298 |
| **C-2** | 0.00270 | 0.0446 | 0.00156 | 0.0335 | 23.083 | 0.350 | 0.900 |
| **C-3** | 0.00287 | 0.0643 | 0.00139 | 0.0223 | 28.000 | 0.536 | 0.906 |
| **0-1** | 0.00350 | 0.0647 | 0.00152 | 0.0140 | 16.750 | 0.274 | 0.721 |
| **0-2** | 0.00513 | 0.0417 | 0.00104 | 0.0257 | 28.667 | 0.442 | 1.247 |
| **0-3** | 0.00570 | 0.0694 | 0.00162 | 0.0262 | 33.333 | 0.537 | 1.088 |
| **3-1** | 0.00313 | 0.0554 | 0.00111 | 0.0185 | 17.417 | 0.395 | 0.787 |
| **3-2** | 0.00467 | 0.0565 | 0.00124 | 0.0165 | 10.917 | 0.363 | 0.726 |
| **3-3** | 0.00347 | 0.0377 | 0.00151 | 0.0325 | 29.833 | 0.440 | 0.846 |
| **6-1** | 0.00490 | 0.0373 | 0.000723 | 0.0269 | 17.500 | 0.354 | 0.859 |
| **6-2** | 0.00503 | 0.0412 | 0.00108 | 0.0405 | 21.500 | 0.492 | 0.987 |
| **6-3** | 0.00347 | 0.0564 | 0.00139 | 0.0380 | 19.000 | 0.422 | 1.051 |
| **12-1** | 0.0138 | 0.0820 | 0.00164 | 0.0578 | 19.167 | 0.430 | 0.512 |
| **12-2** | 0.00990 | 0.0721 | 0.00183 | 0.0613 | 17.583 | 0.356 | 0.873 |
| **12-3** | 0.0162 | 0.0805 | 0.00197 | 0.0459 | 25.667 | 0.346 | 0.741 |
| **24-1** | 0.0103 | 0.0657 | 0.00211 | 0.0287 | 9.917 | 0.462 | 0.812 |
| **24-2** | 0.0143 | 0.0783 | 0.00203 | 0.0349 | 11.500 | 0.299 | 0.504 |
| **24-3** | 0.00843 | 0.0852 | 0.00174 | 0.0410 | 8.000 | 0.394 | 0.846 |
| **36-1** | 0.00457 | 0.0614 | 0.00186 | 0.0207 | 5.500 | 0.355 | 0.504 |
| **36-2** | 0.00853 | 0.0275 | 0.00159 | 0.0257 | 5.750 | 0.446 | 0.622 |
| **36-3** | 0.00557 | 0.0303 | 0.00167 | 0.0376 | 8.250 | 0.383 | 0.529 |
| **48-1** | 0.00347 | 0.0262 | 0.00159 | 0.0342 | 10.333 | 0.439 | 0.599 |
| **48-2** | 0.0111 | 0.0259 | 0.00199 | 0.0256 | 10.667 | 0.404 | 0.644 |
| **48-3** | 0.00620 | 0.0346 | 0.00198 | 0.0326 | 13.083 | 0.437 | 0.881 |
| **PTC-1** | - | 0.0646 | - | - | - | - | - |
| **PTC-2** | - | 0.0616 | - | - | - | - | - |
| **PTC-3** | - | 0.0723 | - | - | - | - | - |
| **STD-1** | - | - | - | 0.000133 | - | 0.0860 | 0.0860 |
| **STD-2** | - | - | - | 0.00430 | - | 0.345 | 0.345 |
| **STD-3** | - | - | - | 0.00527 | - | 0.410 | 0.410 |
| **STD-4** | - | - | - | 0.00937 | - | 0.820 | 0.820 |
| **STD-5** | - | - | - | 0.0193 | - | 1.238 | 1.238 |
| **STD-6** | - | - | - | 0.0328 | - | - | - |
| **STD-7** | - | - | - | 0.0598 | - | - | - |

Treatment (Groups) Format: A-B (A: Time Point Post-AHPND Infection/Uninfected Control (C)/Positive Control (PTC)/Standard (STD); B: Number of Biological Replicate)

PO, RB, SOD, Nitrite, Ca^2+^, Bradford: OD Biological Values

THC: Average Total Cells

HP: Hepatopancreas

Nitrite Standard: 1.56, 3.13, 6.25, 12.5, 25.0, 50.0, 100.0 µM

Ca^2+^ Standard: 1.0 mmol/L

Bradford Standard: 0.125, 0.75, 1.0, 2.0, 3.0 mg/ml
