# Supplementary material for: Biochemical indexes and gut microbiota testing as diagnostic methods for Penaeus monodon health and physiological changes during AHPND infection with food safety concerns
Source: Food Sci Nutr. 2022 Apr 22;10(8):2694–709. doi: 10.1002/fsn3.2873 (PMC9361443; doi:10.1002/fsn3.2873)
Supplement: Supplementary file 2 — Figure S1 [file FSN3-10-2694-s012.docx]

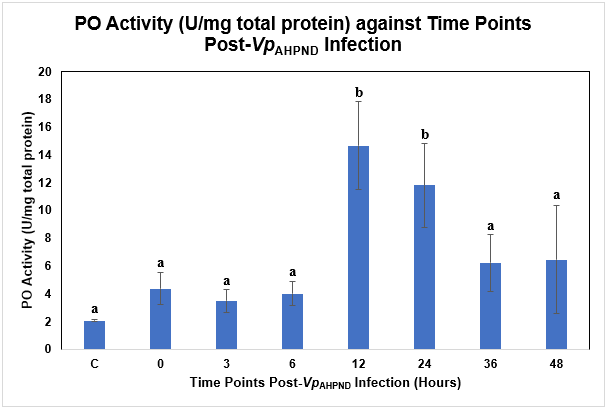


**Figure 1 Supp: Phenoloxidase (PO) Activity (U/mg total protein) against Time Post-*Vp*_AHPND_ Infection (Hours) using *Vp*_AHPND_-infected *P. monodon* hepatopancreas tissue samples.**

C= Uninfected Control

a and b represent different subsets obtained in Duncan post hoc test.

The error bars indicated standard deviations of the data.
