# Supplementary figures and images for: Biochemical indexes and gut microbiota testing as diagnostic methods for Penaeus monodon health and physiological changes during AHPND infection with food safety concerns
Source: Food Sci Nutr. 2022 Apr 22;10(8):2694–709. doi: 10.1002/fsn3.2873 (PMC9361443; doi:10.1002/fsn3.2873)

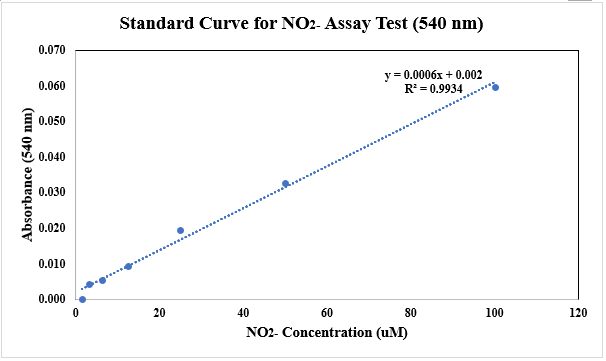


**Figure 4 Supp: Standard Curve of Nitrite (NO2-) (OD 540 nm) Assay Test.**

Supplement: Supplementary file 5 — Figure S4 [file FSN3-10-2694-s015.docx]

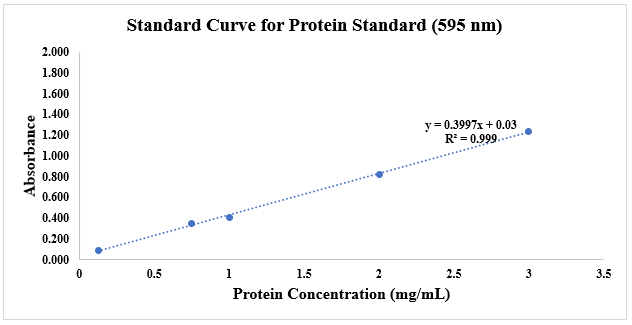


**Figure 7 Supp: Bradford’s Test standard curve plotted using protein BSA standard (595 nm).**

Supplement: Supplementary file 8 — Figure S7 [file FSN3-10-2694-s014.docx]
