# Supplementary material for: Biochemical indexes and gut microbiota testing as diagnostic methods for Penaeus monodon health and physiological changes during AHPND infection with food safety concerns
Source: Food Sci Nutr. 2022 Apr 22;10(8):2694–709. doi: 10.1002/fsn3.2873 (PMC9361443; doi:10.1002/fsn3.2873)
Supplement: Supplementary file 9 — Figure S8 [file FSN3-10-2694-s016.docx]

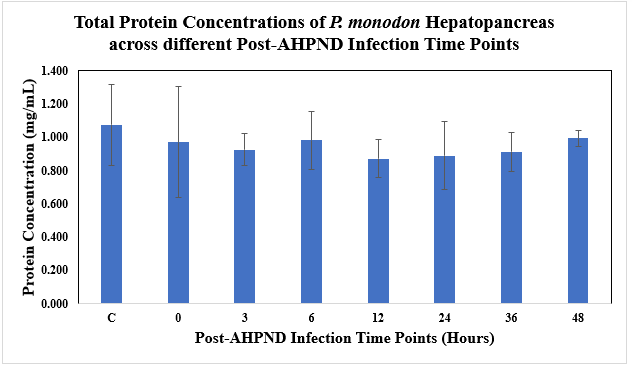


**Figure 8 Supp: Total protein concentrations of *Vp*_AHPND_-infected *P. monodon* hepatopancreas samples at different post-infection time points determined using Bradford’s Test standard curve plotted (595 nm).**

C= Uninfected Control
