# Supplementary material for: Biochemical indexes and gut microbiota testing as diagnostic methods for Penaeus monodon health and physiological changes during AHPND infection with food safety concerns
Source: Food Sci Nutr. 2022 Apr 22;10(8):2694–709. doi: 10.1002/fsn3.2873 (PMC9361443; doi:10.1002/fsn3.2873)
Supplement: Supplementary file 10 — Figure S9 [file FSN3-10-2694-s013.docx]

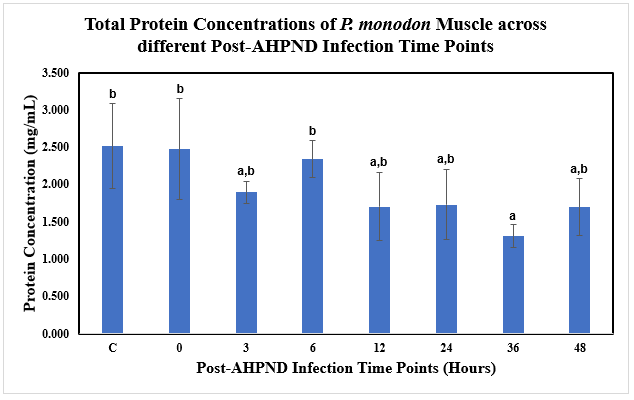


**Figure 9 Supp: Total protein concentrations of *Vp*_AHPND_-infected *P. monodon* muscle samples at different post-infection time points determined using Bradford’s Test standard curve plotted (595 nm).**

C= Uninfected Control

a and b represent different subsets obtained in Duncan post hoc test.

The error bars indicated standard deviations of the data.
