# Supplementary material for: Biochemical indexes and gut microbiota testing as diagnostic methods for Penaeus monodon health and physiological changes during AHPND infection with food safety concerns
Source: Food Sci Nutr. 2022 Apr 22;10(8):2694–709. doi: 10.1002/fsn3.2873 (PMC9361443; doi:10.1002/fsn3.2873)
Supplement: Supplementary file 11 — Figure S10 [file FSN3-10-2694-s003.docx]

**Figure 10 Supp: Rarefaction analysis of uninfected control (CTL) and *Vp*_AHPND_-infected (APM) *P. monodon* gut sample 16S analysis results.**
