# Supplementary material for: Biochemical indexes and gut microbiota testing as diagnostic methods for Penaeus monodon health and physiological changes during AHPND infection with food safety concerns
Source: Food Sci Nutr. 2022 Apr 22;10(8):2694–709. doi: 10.1002/fsn3.2873 (PMC9361443; doi:10.1002/fsn3.2873)
Supplement: Supplementary file 13 — Figure S12 [file FSN3-10-2694-s020.docx]

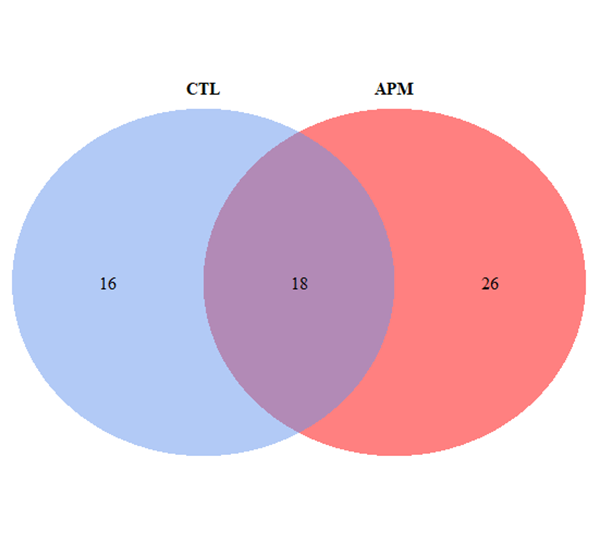


**Figure 12 Supp: Venn diagram distribution (distance= 0.03) of OTUs from uninfected control group (CTL) and *Vp*_AHPND_-infected group (APM).**
