# Supplementary material for: Biochemical indexes and gut microbiota testing as diagnostic methods for Penaeus monodon health and physiological changes during AHPND infection with food safety concerns
Source: Food Sci Nutr. 2022 Apr 22;10(8):2694–709. doi: 10.1002/fsn3.2873 (PMC9361443; doi:10.1002/fsn3.2873)
Supplement: Supplementary file 14 — Table S1 [file FSN3-10-2694-s018.docx]

**Table 1 Supp: Statistical validation of (A) One-Way ANOVA Analysis and (B) Post-hoc Duncan Test for Phenoloxidase (PO) Activity (U/mg total protein) against Time Points Post-*Vp*_AHPND_ Infection (Hours).**

A)

| **ANOVA** | | | | | |
| --- | --- | --- | --- | --- | --- |
| **PO Activity (U/mg total protein)** | | | | | |
|  | **Sum of Squares** | **df** | **Mean Square** | **F** | **Sig.** |
| Between Groups | 402.675 | 7 | 57.525 | 9.538 | <0.001 |
| Within Groups | 96.499 | 16 | 6.031 |  |  |
| Total | 499.174 | 23 |  |  |  |

B)

| **PO Activity (U/mg total protein)** | | | |
| --- | --- | --- | --- |
| **Duncan^a^** | | | |
| **Time Post-*Vp*_AHPND_ Infection (Hours)** | **N** | **Subset for alpha = 0.05** | |
|  |  | **a** | **b** |
| C | 3 | 2.06940 |  |
| 3 | 3 | 3.48370 |  |
| 6 | 3 | 4.00481 |  |
| 0 | 3 | 4.37544 |  |
| 36 | 3 | 6.23798 |  |
| 48 | 3 | 6.44894 |  |
| 24 | 3 |  | 11.80794 |
| 12 | 3 |  | 14.66461 |
| Sig. |  | 0.068 | 0.173 |
| Means for groups in homogeneous subsets are displayed. | | | |
| a. Uses Harmonic Mean Sample Size = 3.000. | | | |
