# Supplementary material for: Biochemical indexes and gut microbiota testing as diagnostic methods for Penaeus monodon health and physiological changes during AHPND infection with food safety concerns
Source: Food Sci Nutr. 2022 Apr 22;10(8):2694–709. doi: 10.1002/fsn3.2873 (PMC9361443; doi:10.1002/fsn3.2873)
Supplement: Supplementary file 15 — Table S2 [file FSN3-10-2694-s017.docx]

**Table 2 Supp: Statistical validation of (A) One-Way ANOVA Analysis and (B) Post-hoc Duncan Test for Respiratory Burst (RB) (OD 630 nm) against Time Points Post-*Vp*_AHPND_ Infection (Hours).**

A)

| **ANOVA** | | | | | |
| --- | --- | --- | --- | --- | --- |
| **RB (OD 630 nm)** | | | | | |
|  | **Sum of Squares** | **df** | **Mean Square** | **F** | **Sig.** |
| Between Groups | 0.007 | 8 | 0.001 | 6.354 | 0.001 |
| Within Groups | 0.002 | 18 | 0.000 |  |  |
| Total | 0.009 | 26 |  |  |  |

B)

| **RB (OD 630 nm)** | | | | | |
| --- | --- | --- | --- | --- | --- |
| **Duncan^a^** | | | | | |
| **Time Post-*Vp*_AHPND_ Infection (Hours)** | **N** | **Subset for alpha = 0.05** | | | |
|  |  | **a** | **b** | **c** | **d** |
| 48 | 3 | 0.028894 |  |  |  |
| 36 | 3 | 0.039717 | 0.039717 |  |  |
| 6 | 3 | 0.044939 | 0.044939 | 0.044939 |  |
| C | 3 | 0.048339 | 0.048339 | 0.048339 |  |
| 3 | 3 | 0.049839 | 0.049839 | 0.049839 |  |
| 0 | 3 |  | 0.058572 | 0.058572 | 0.058572 |
| PTC | 3 |  |  | 0.066172 | 0.066172 |
| 24 | 3 |  |  |  | 0.076394 |
| 12 | 3 |  |  |  | 0.078161 |
| Sig. |  | 0.057 | 0.084 | 0.054 | 0.068 |
| Means for groups in homogeneous subsets are displayed. | | | | | |
| a. Uses Harmonic Mean Sample Size = 3.000. | | | | | |
