# Supplementary material for: Biochemical indexes and gut microbiota testing as diagnostic methods for Penaeus monodon health and physiological changes during AHPND infection with food safety concerns
Source: Food Sci Nutr. 2022 Apr 22;10(8):2694–709. doi: 10.1002/fsn3.2873 (PMC9361443; doi:10.1002/fsn3.2873)
Supplement: Supplementary file 16 — Table S3 [file FSN3-10-2694-s010.docx]

**Table 3 Supp: Statistical validation of (A) One-Way ANOVA Analysis and (B) Post-hoc Duncan Test for Average Relative SOD Activity against Time Points Post-*Vp*_AHPND_ Infection (Hours).**

A)

| **ANOVA** | | | | | |
| --- | --- | --- | --- | --- | --- |
| **Average Relative SOD Activity** | | | | | |
|  | **Sum of Squares** | **df** | **Mean Square** | **F** | **Sig.** |
| Between Groups | 14115.995 | 7 | 2016.571 | 6.034 | 0.001 |
| Within Groups | 5346.949 | 16 | 334.184 |  |  |
| Total | 19462.944 | 23 |  |  |  |

B)

| **Average Relative SOD Activity** | | | | | |
| --- | --- | --- | --- | --- | --- |
| **Duncan^a^** | | | | | |
| **Time Post-*Vp*_AHPND_ Infection (Hours)** | **N** | **Subset for alpha = 0.05** | | | |
|  |  | **a** | **b** | **c** | **d** |
| 24 | 3 | 26.26 |  |  |  |
| 48 | 3 | 35.03 | 35.03 |  |  |
| 12 | 3 | 38.50 | 38.50 |  |  |
| 36 | 3 | 47.00 | 47.00 | 47.00 |  |
| C | 3 |  | 68.83 | 68.83 | 68.83 |
| 0 | 3 |  |  | 72.98 | 72.98 |
| 3 | 3 |  |  |  | 81.85 |
| 6 | 3 |  |  |  | 100.00 |
| Sig. |  | 0.219 | 0.052 | 0.117 | 0.071 |
| Means for groups in homogeneous subsets are displayed. | | | | | |
| a. Uses Harmonic Mean Sample Size = 3.000. | | | | | |
