# Supplementary material for: Biochemical indexes and gut microbiota testing as diagnostic methods for Penaeus monodon health and physiological changes during AHPND infection with food safety concerns
Source: Food Sci Nutr. 2022 Apr 22;10(8):2694–709. doi: 10.1002/fsn3.2873 (PMC9361443; doi:10.1002/fsn3.2873)
Supplement: Supplementary file 17 — Table S4 [file FSN3-10-2694-s019.docx]

**Table 4 Supp: Statistical validation of (A) One-Way ANOVA Analysis and (B) Post-hoc Duncan Test for Nitrite (NO2-) Concentration (OD 540 nm) against Time Points Post-*Vp*_AHPND_ Infection (Hours).**

A)

| **ANOVA** | | | | | |
| --- | --- | --- | --- | --- | --- |
| **NO_2-_ Concentration (nmol/ml)** | | | | | |
|  | **Sum of Squares** | **df** | **Mean Square** | **F** | **Sig.** |
| Between Groups | 6459.695 | 7 | 922.814 | 6.536 | 0.001 |
| Within Groups | 2259.163 | 16 | 141.198 |  |  |
| Total | 8718.858 | 23 |  |  |  |

B)

| **NO_2-_ Concentration (uM)** | | | |
| --- | --- | --- | --- |
| **Duncan^a^** | | | |
| **Time Post-*Vp*_AHPND_ Infection (Hours)** | **N** | **Subset for alpha = 0.05** | |
|  |  | **a** | **b** |
| 0 | 3 | 33.2778 |  |
| 3 | 3 | 34.1481 |  |
| C | 3 | 42.9444 |  |
| 36 | 3 | 43.2963 |  |
| 48 | 3 | 48.0000 |  |
| 24 | 3 | 54.7963 |  |
| 6 | 3 | 55.1852 |  |
| 12 | 3 |  | 88.3889 |
| Sig. |  | 0.062 | 1.000 |
| Means for groups in homogeneous subsets are displayed. | | | |
| a. Uses Harmonic Mean Sample Size = 3.000. | | | |
