# Supplementary material for: Biochemical indexes and gut microbiota testing as diagnostic methods for Penaeus monodon health and physiological changes during AHPND infection with food safety concerns
Source: Food Sci Nutr. 2022 Apr 22;10(8):2694–709. doi: 10.1002/fsn3.2873 (PMC9361443; doi:10.1002/fsn3.2873)
Supplement: Supplementary file 18 — Table S5 [file FSN3-10-2694-s006.docx]

**Table 5 Supp: Statistical validation of (A) One-Way ANOVA Analysis and (B) Post-hoc Duncan Test for THC (x10^5 ml^-1^) against Time Points Post-*Vp*_AHPND_ Infection (Hours).**

A)

| **ANOVA** | | | | | |
| --- | --- | --- | --- | --- | --- |
| **THC (x10^5 ml^-1^)** | | | | | |
|  | **Sum of Squares** | **df** | **Mean Square** | **F** | **Sig.** |
| Between Groups | 42.269 | 7 | 6.038 | 5.756 | 0.002 |
| Within Groups | 16.785 | 16 | 1.049 |  |  |
| Total | 59.054 | 23 |  |  |  |

B)

| **THC (x10^5 ml^-1^)** | | | | |
| --- | --- | --- | --- | --- |
| **Duncan^a^** | | | | |
| **Time Post-*Vp*_AHPND_ Infection (Hours)** | **N** | **Subset for alpha = 0.05** | | |
|  |  | **a** | **b** | **c** |
| 0 | 3 | 5.2500 |  |  |
| C | 3 | 4.7778 |  |  |
| 12 | 3 | 4.1611 | 4.1611 |  |
| 3 | 3 | 3.8778 | 3.8778 |  |
| 6 | 3 | 3.8667 | 3.8667 |  |
| 48 | 3 |  | 2.2722 | 2.2722 |
| 24 | 3 |  |  | 1.9611 |
| 36 | 3 |  |  | 1.3000 |
| Sig. |  | 0.154 | 0.053 | 0.287 |
| Means for groups in homogeneous subsets are displayed. | | | | |
| a. Uses Harmonic Mean Sample Size = 3.000. | | | | |
