# Supplementary material for: Biochemical indexes and gut microbiota testing as diagnostic methods for Penaeus monodon health and physiological changes during AHPND infection with food safety concerns
Source: Food Sci Nutr. 2022 Apr 22;10(8):2694–709. doi: 10.1002/fsn3.2873 (PMC9361443; doi:10.1002/fsn3.2873)
Supplement: Supplementary file 19 — Table S6 [file FSN3-10-2694-s009.docx]

**Table 6 Supp: One-Way Analysis of Variance (One-Way ANOVA) showing statistical significance of *P. monodon* hepatopancreas total protein concentrations post-AHPND infection obtained.**

| **ANOVA** | | | | | |
| --- | --- | --- | --- | --- | --- |
| **Protein Concentration (mg/mL)** | | | | | |
|  | **Sum of Squares** | **df** | **Mean Square** | **F** | **Sig.** |
| Between Groups | 0.093 | 7 | 0.013 | 0.377 | 0.902 |
| Within Groups | 0.561 | 16 | 0.035 |  |  |
| Total | 0.653 | 23 |  |  |  |
