# Supplementary material for: Biochemical indexes and gut microbiota testing as diagnostic methods for Penaeus monodon health and physiological changes during AHPND infection with food safety concerns
Source: Food Sci Nutr. 2022 Apr 22;10(8):2694–709. doi: 10.1002/fsn3.2873 (PMC9361443; doi:10.1002/fsn3.2873)
Supplement: Supplementary file 20 — Table S7 [file FSN3-10-2694-s001.docx]

**Table 7 Supp: Statistical validation of (A) One-Way Analysis of Variance (One-Way ANOVA) and (B) post-hoc Duncan test for *P. monodon* muscle total protein concentrations post-AHPND infection obtained.**

**A)**

| **ANOVA** | | | | | |
| --- | --- | --- | --- | --- | --- |
| **Protein Concentration (mg/mL)** | | | | | |
|  | **Sum of Squares** | **df** | **Mean Square** | **F** | **Sig.** |
| Between Groups | 4.018 | 7 | 0.574 | 3.143 | 0.027 |
| Within Groups | 2.922 | 16 | 0.183 |  |  |
| Total | 6.940 | 23 |  |  |  |

**B)**

| **Protein Concentration (mg/mL)** | | | |
| --- | --- | --- | --- |
| **Duncan^a^** | | | |
| **Time Post-AHPND Infection (Hours)** | **N** | **Subset for alpha = 0.05** | |
|  |  | **a** | **b** |
| 36 | 3 | 1.30510 |  |
| 48 | 3 | 1.69640 | 1.69640 |
| 12 | 3 | 1.69790 | 1.69790 |
| 24 | 3 | 1.72867 | 1.72867 |
| 3 | 3 | 1.89284 | 1.89284 |
| 6 | 3 |  | 2.34096 |
| 0 | 3 |  | 2.47348 |
| C | 3 |  | 2.51284 |
| Sig. |  | 0.147 | 0.054 |
| Means for groups in homogeneous subsets are displayed. | | | |
| a. Uses Harmonic Mean Sample Size = 3.000. | | | |
