# Supplementary material for: Biochemical indexes and gut microbiota testing as diagnostic methods for Penaeus monodon health and physiological changes during AHPND infection with food safety concerns
Source: Food Sci Nutr. 2022 Apr 22;10(8):2694–709. doi: 10.1002/fsn3.2873 (PMC9361443; doi:10.1002/fsn3.2873)
Supplement: Supplementary file 21 — Table S8 [file FSN3-10-2694-s007.docx]

**Table 8 Supp: The alpha diversity parameters of 16S rRNA sequencing data.**

| **Treatment Group** | **Good’s Coverage** | **Chao 1** | **Shannon** | **Simpson** |
| --- | --- | --- | --- | --- |
| **CTL** | 0.9999 | 78.50  (LCI: 78.040, HCI: 84.213) | 2.286  (LCI: 1.925, HCI: 2.646) | 0.201  (LCI: 0.198, HCI: 0.204) |
| **APM** | 0.9999 | 91.857  (LCI: 91.092, HCI: 98.973) | 2.912  (LCI: 2.592, HCI: 3.232) | 0.327  (LCI: 0.322, HCI: 0.332) |

LCI= Minimum Value, HCI= Maximum Value

CTL= Uninfected Control, APM= *Vp*_AHPND_-infected
